# Targeting STE20-type kinase MST3 improves metabolic dysfunction-associated steatohepatitis without affecting hepatocellular carcinoma development in mice

**DOI:** 10.1186/s12916-026-04812-0

**Published:** 2026-03-24

**Authors:** Jingjing Zhang, Xiangdong Gongye, Lohitesh Kovooru, Emma Andersson, Bernice Asiedu, Manoj Amrutkar, Nadia Gul, Caitlyn Myers, Sheri Booten, Dan Emil Lind, Ying Xia, Antonio Molinaro, Anetta Härtlova, Per Lindahl, Sue Murray, Margit Mahlapuu

**Affiliations:** 1 Department of Chemistry and Molecular Biology, University of Gothenburg and Sahlgrenska University Hospital, Gothenburg, Sweden; 2 Department of Molecular and Clinical Medicine, Institute of Medicine, University of Gothenburg, Gothenburg, Sweden; 3 Wallenberg Centre for Molecular and Translational Medicine, University of Gothenburg, Gothenburg, Sweden; 4 Department of Pathology, Oslo University Hospital Rikshospitalet, Oslo, Norway; 5 Sahlgrenska Center for Cancer Research, Department of Medical Biochemistry and Cell Biology, Institute of Biomedicine, University of Gothenburg, Gothenburg, Sweden; 6 Department of Microbiology and Immunology, Institute of Biomedicine, University of Gothenburg, Gothenburg, Sweden; 7 Ionis Pharmaceuticals, Carlsbad, USA; 8 Department of Medical Biochemistry and Cell Biology, Institute of Biomedicine, University of Gothenburg and Sahlgrenska University Hospital, Gothenburg, Sweden; 9 Department of Liver Surgery, Renji Hospital, Shanghai Jiao Tong University School of Medicine, Shanghai, China; 10 Department of Medicine, Sahlgrenska University Hospital, Gothenburg, Sweden; 11 Institute of Medical Microbiology and Hygiene, Medical Center – University of Freiburg, Freiburg, Germany

**Keywords:** MST3, Antisense oligonucleotide therapy, Hepatocellular carcinoma, Metabolic dysfunction-associated steatohepatitis

## Abstract

**Background:**

Metabolic dysfunction-associated steatohepatitis (MASH) is a major precursor of hepatocellular carcinoma (HCC), yet the molecular mechanisms linking steatohepatitis to malignancy remain poorly defined. The STE20-type kinase MST3 associates with hepatocellular lipid droplets and regulates metabolic homeostasis and stress responses in the liver. Here, we investigated whether pharmacologic inhibition of MST3 could attenuate the initiation and progression of MASH-associated HCC in vivo.

**Methods:**

The therapeutic potential of MST3 inhibition was evaluated in a mouse model in which MASH-HCC was induced by a single diethylnitrosamine injection followed by 30 weeks of Western-style diet feeding. Liver tumor burden was assessed after 12, 21, or 30 weeks of treatment with *Mst3*-targeting antisense oligonucleotide (ASO) or a non-targeting control ASO, and histological, biochemical, and mechanistic analyses were performed in the 30-week cohort. In parallel, proteomic profiling of CRISPR/Cas9-generated *MST3* knockout and wild-type Huh7 cells was conducted to gain molecular insight into MST3-regulated pathways.

**Results:**

*Mst3* ASO therapy had no impact on the onset or aggravation of experimentally induced MASH-associated HCC in mice, despite markedly improving the whole-body metabolic profile and suppressing all key features of MASH. Proteomic profiling of MST3-deficient hepatocytes revealed coordinated activation of mitochondrial and lysosomal pathways, consistent with enhanced fatty acid degradation and catabolic clearance.

**Conclusions:**

Our findings in the selected mouse model of MASH-HCC suggest that MST3 is dispensable for hepatocarcinogenesis, yet its antagonism dampens diet-induced metabolic dysfunction and effectively attenuates MASH severity, challenging the prevailing assumption that targeting MASH driver genes alone is sufficient to prevent HCC development in the context of obesity.

**Supplementary Information:**

The online version contains supplementary material available at 10.1186/s12916-026-04812-0.

## Background

Metabolic dysfunction-associated steatotic liver disease (MASLD) encompasses a spectrum of obesity-related liver disorders, ranging from benign hepatic steatosis (defined by the accumulation of triglycerides in more than 5% of hepatocytes) to metabolic dysfunction-associated steatohepatitis (MASH; characterized by liver steatosis accompanied by inflammation, hepatocellular injury, and potentially fibrosis) [1, 2]. Driven by shifts in diet and lifestyle, the prevalence of MASLD has steadily increased over the past three decades and now affects more than 30% of the global population [3, 4]. Notably, about 20% of MASLD patients progress to MASH, and a subset of these subjects (approximately 2%) subsequently develop MASH-associated hepatocellular carcinoma (MASH-HCC) [5, 6]. HCC is currently the third leading cause of cancer-related death worldwide and exhibits the fastest rising incidence and mortality rates among all tumor types [7–9]. Historically, the primary risk factors for HCC have included chronic infections with hepatitis B and C viruses and excessive alcohol consumption [10, 11]. However, with the growing prevalence of MASLD and improved efficacy of viral hepatitis therapies, MASH has emerged as a significant contributor to the global burden of HCC [12, 13].

Our recent studies provide multiple lines of translational evidence supporting a critical role for MST3 (mammalian sterile 20-like 3; also known as STK24), MST4 (mammalian sterile 20-like 4; also known as STK26 or MASK), and STK25 (serine/threonine protein kinase 25; also known as YSK1 or SOK1) as key molecular drivers of hepatocellular steatotoxicity [14]. These three proteins comprise the GCKIII subfamily of STE20-type kinases and are all localized to the surface of intrahepatocellular lipid droplets [15–19]. Interestingly, we found that *MST3, MST4*, and *STK25* expression in human liver biopsies correlates positively with the severity of MASH, including hepatic lipid content, inflammation, and cell injury (ballooning) [16, 17, 20]. We also reported that silencing of MST3, MST4, or STK25 in human hepatocytes cell-autonomously suppresses ectopic fat deposition by shifting metabolism from lipid anabolism toward catabolism [15–17, 21]. Moreover, hepatocytes deficient in any of these kinases display substantial resistance to lipotoxicity-triggered oxidative and endoplasmic reticulum (ER) stress—two main contributors to disease progression from simple liver steatosis to MASH [16, 17, 20, 21]. Conversely, in vitro overexpression of GCKIII kinases markedly escalates hepatocellular lipid deposition and exacerbates oxidative and ER stress [17, 20, 21]. In vivo, both genetic and pharmacologic inhibition of MST3 or STK25 in mice confers robust protection against all major features of diet-induced MASH, including reductions in hepatic steatosis, lobular inflammation, and nutritional fibrosis [20, 22–26]. In contrast, no differences were observed in the onset or aggravation of MASH in obese *Mst4* knockout mice when compared with wild-type controls [27].

Importantly, by analyzing public databases and in-house cohorts, we found that gene expression of *MST3*, *MST4*, and *STK25* is significantly higher in human HCC tissues compared with nontumor controls [28, 29]. Furthermore, in vitro silencing of MST3, MST4, or STK25 suppresses tumorigenic properties of human HCC cells, including markedly decreased proliferation, migration, invasion, and epithelial-mesenchymal transition [28–30]. Notably, in vivo inhibition of STK25 has been shown to efficiently mitigate the onset and progression of hepatocarcinogenesis in mouse models, in which MASH-HCC was triggered by injections of chemical procarcinogens in combination with a dietary challenge [29, 30]. Collectively, these data support the hypothesis that GCKIII kinases may play an oncogenic role in HCC development in the context of MASH.

Building on these earlier findings, we examined whether antagonizing MST3 activity in vivo could not only attenuate MASH but also block its transition to HCC. To test this, we conducted a proof-of-principle experiment evaluating the efficacy of *Mst3*-targeting antisense oligonucleotide (ASO) in preventing the initiation and halting the progression of MASH-associated HCC in mice when administered at different stages of disease development. Interestingly, we found that treatment with *Mst3* ASO had no impact on HCC tumor burden, despite robust improvement in whole-body metabolic profile and suppression of MASH severity.

## Methods

### Animal experiments

**Fig. 1:**
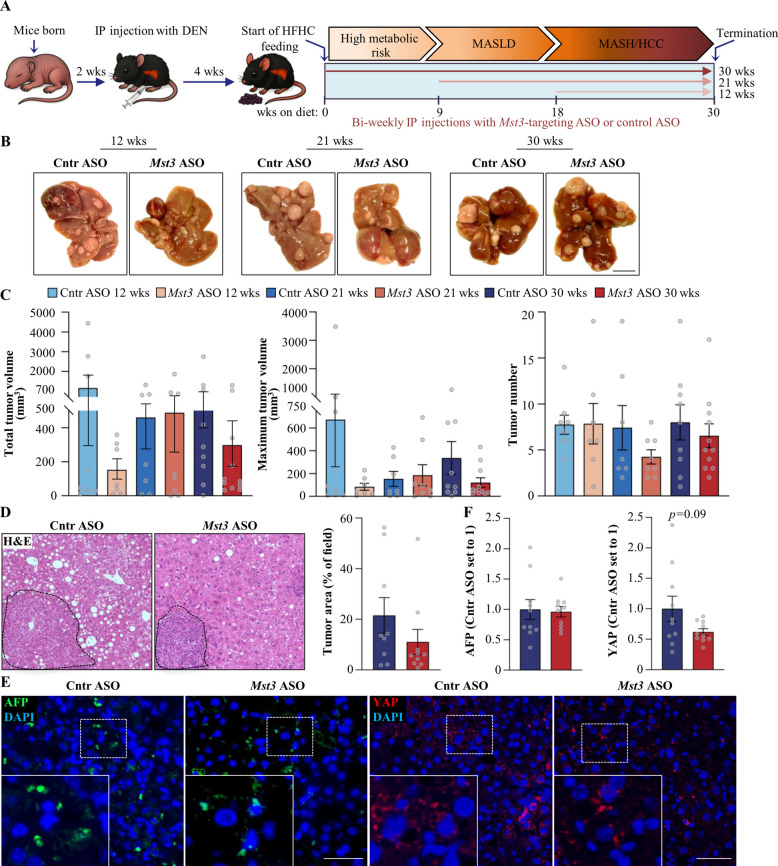
*Mst3* ASO therapy does not affect the initiation or progression of experimentally induced HCC in obese mice. **A** Schematic overview of the experimental design. **B** Representative images of whole livers. Scale bar: 5 mm. **C** Quantification of total tumor volume, maximum tumor volume, and tumor number on the liver surface. **D** Representative images of liver sections stained with H&E. Scale bar: 100 μm. Quantification of tumor area. **E** Representative images of liver sections processed for immunofluorescence with anti-AFP (green) or anti-YAP (red) antibodies; nuclei stained with DAPI (blue). Scale bar: 100 μm. **F** Quantification of fluorescence-positive area. Liver samples analyzed in **D**–**F** were collected from mice treated with *Mst3* ASO or control ASO for 30 weeks. Data are mean ± SEM from 7 to 11 mice per group. Cntr, control; HFHC, high-fat, high-cholesterol diet; IP, intraperitoneal; wks, weeks

Male C57BL/6 J mice (RRID: IMSR_JAX:000664; Charles River, Sulzfeld, Germany) were housed 3–5 per cage in a temperature-controlled facility (21 °C) under a 12-h light/dark cycle and ad libitum access to chow and water. To induce HCC in the context of MASH, a single intraperitoneal injection of diethylnitrosamine (DEN; 25 mg/kg; N0258; Sigma-Aldrich, St. Louis, MO) was given to 2-week-old mice. Four weeks after DEN injection, mice were switched to a Western-style diet (40 kcal% fat, primarily palm oil, 20 kcal% fructose, and 2 kcal% cholesterol; D09100310; Research Diets, New Brunswick, NJ) for 30 weeks, which is a regimen previously validated to promote MASH-associated HCC [31]. At 6 weeks of age, the weight-matched mice were randomized to intraperitoneal injections of *Mst3*-targeting ASO (50 mg/kg/week) or a non-targeting control ASO (50 mg/kg/week) [26], twice weekly for the last 12, 21, or 30 weeks of Western-style diet feeding (see Fig. 1A for a schematic overview of the experimental design). Body weights were recorded weekly, and at the indicated time points, blood samples were collected from the tail vein under fasting conditions to determine plasma glucose and insulin levels. At the age of 36 weeks, mice were sacrificed by cervical dislocation under isoflurane anesthesia after a 4-h fast. Blood was obtained by cardiac puncture for assessment of alanine aminotransferase (ALT) levels. Livers were weighed, each lobe was photographed, and the surface tumor parameters were evaluated as previously reported [30]. Liver tissues were then processed for flow cytometry, harvested for histological and immunohistochemical/immunofluorescence analysis, or snap frozen in liquid nitrogen and stored at − 80 °C for examination of protein and gene expression, and biochemical parameters, as described below.

The mice in this study were cared for in accordance with the National Institutes of Health (NIH; Bethesda, MD) guidelines as outlined in the *Guide for the Care and Use of Laboratory Animals*. All the in vivo experiments adhered to protocols approved by the local Ethics Committee for Animal Studies at the Administrative Court of Appeals in Gothenburg, Sweden (approval number 5.8.18–20691/2023).

### In vivo tests

Body composition analysis (BCA) of total, fat, and lean body mass was carried out by time-domain nuclear magnetic resonance (TD-NMR) using a Minispec LF110 Analyzer (Bruker Corporation, Rheinstetten, Germany). Food intake was measured 2–3 times during the study over 48-h periods and reported as average consumption per 24 h. Glucose and insulin tolerance tests (GTT and ITT) were conducted after a 4-h fast via intraperitoneal injection of glucose (1 g/kg; Sigma-Aldrich) or human recombinant insulin (1 U/kg; Actrapid Penfill; Novo Nordisk, Bagsværd, Denmark), respectively.

### Histological, immunohistochemical, and immunofluorescence evaluation

Mouse liver tissues were fixed in 4% (vol/vol) phosphate-buffered formaldehyde (Histolab Products, Gothenburg, Sweden), embedded in paraffin, and sectioned. Paraffin sections were stained with hematoxylin and eosin (H&E) or Picrosirius Red (Histolab Products) for morphological assessment. The MASLD activity score (MAS) and the fibrosis stage were determined according to the Kleiner/Brunt criteria adapted for use in rodents [32, 33]. The scoring of Mallory-Denk bodies (MDBs) was conducted according to the Goodman classification [34]. For immunohistochemical and immunofluorescence analysis, sections were incubated with primary antibodies, followed by incubation with biotinylated or fluorescently labeled secondary antibodies (see Additional File 1: Supplementary Table S1 for antibody details). Liver tissue samples were also embedded in optimal cutting temperature (OCT) mounting medium (Histolab Products) and frozen in liquid nitrogen for cryosectioning. Cryosections were stained with Bodipy 493/503 (Invitrogen, Carlsbad, CA) to visualize neutral lipids or Mitotracker Red (Thermo Fisher Scientific, Waltham, MA) to monitor mitochondrial membrane potential. Images were captured using a Zeiss Axio Observer microscope with ZEN Blue software (Zeiss, Oberkochen, Germany). The total area labeled was quantified in 6–8 randomly selected microscopic fields (× 100 or × 200; distributed over three non-consecutive sections) per mouse using the ImageJ software (RRID: SCR_003070; NIH).

### Biochemical assays

Fasting blood glucose and plasma insulin were measured using the Accu-Chek glucometer (Roche, Basel, Switzerland) and the Ultra-Sensitive Mouse Insulin ELISA Kit (Crystal Chem, Downers Grove, IL), respectively. Hepatic glycogen levels were quantified from liver lysates using the Glycogen Assay Kit (Sigma-Aldrich) and plasma ALT concentrations were determined using the Alanine Aminotransferase Activity Assay Kit (Sigma-Aldrich). All assays were performed in duplicate.

### Flow cytometry

Fresh mouse liver tissues were digested with 1 mg/mL collagenase IV (Abcam, Cambridge, UK) at 37 °C for 30 min. The suspension was passed through a 100-µm mesh (Thermo Fisher Scientific) and subjected to density-gradient centrifugation using Lymphocyte Separation Medium (MP Biomedicals, Irvine, CA) to obtain liver-infiltrating lymphocytes. Cells were blocked in buffer containing 5 mmol/L EDTA, 1% (wt/vol) bovine serum albumin (BSA), and 0.05% (wt/vol) NaN₃ in PBS, and then stained with fluorophore-conjugated antibodies at 4 °C for 1 h in the dark (see Additional File 1: Supplementary Table S1 for antibody details). Data were acquired on a BD LSRFortessa X-20 (BD Biosciences, San Jose, CA) and analyzed using FlowJo software (RRID: SCR_008520; Tree Star, Ashland, OR).

### CRISPR/Cas9-mediated *MST3* knockout in Huh7 cells and whole-cell proteomics

Details of the experiment are provided in the Additional File 2: Supplementary Methods [35, 36].

### Western blot and RT-qPCR

Western blotting was performed as previously described [37] using the antibodies listed in Additional File 1: Supplementary Table S1. Total RNA was extracted using the E.Z.N.A. Total RNA Kit (Omega Bio-Tek, Norcross, GA), and complementary DNA (cDNA) was synthesized with the High-Capacity cDNA Reverse Transcription Kit (Thermo Fisher Scientific). RT-qPCR was carried out on a CFX Connect Real-Time System (Bio-Rad, Hercules, CA) and target gene expression was normalized to the endogenous control 18S rRNA (Thermo Fisher Scientific).

### Statistical analysis

Quantitative data were analyzed and visualized using GraphPad Prism (GraphPad Software). Pairwise comparisons between two groups were evaluated using two-tailed unpaired Student’s *t*-tests. Statistical significance was defined as *p* < 0.05. All data are presented as mean ± standard error of the mean (SEM), and “*n*” denotes biological replicates, with exact sample sizes specified in the figure legends.

## Results

### *Mst3* ASO therapy has no impact on the initiation or progression of experimentally induced HCC in obese mice

To investigate the efficacy of pharmacological MST3 inhibitors in the prevention and treatment of HCC in a MASH-like context, we employed a mouse model in which MASH-HCC was triggered through a single injection of the chemical procarcinogen DEN, combined with a Western-style diet feeding. *Mst3* ASO (complementary to the 16-nucleotide intronic region of the mouse *Mst3* gene) or control ASO (a non-targeting ASO of the same length and chemistry as *Mst3* ASO) were administered by bi-weekly intraperitoneal injections. ASO treatment was initiated in three groups of mice at different stages of the MASH-related HCC progression timeline: (1) metabolically healthy mice with normal liver fat levels (dosed for 30 weeks); (2) mice exhibiting early-stage MASLD characterized by hepatic steatosis without marked inflammation or fibrosis (dosed for 21 weeks); and (3) mice with established MASH defined by significant hepatic steatosis, inflammation, and fibrosis, but with few or no detectable HCC tumors (dosed for 12 weeks; Fig. 1A).

Consistent with our previous investigations [26], administration of the selected *Mst3* ASO at a dose level of 50 mg/kg/week resulted in > 70% suppression of hepatic MST3 protein abundance (Additional File 3: Supplementary Figure S1*A*). Importantly, no change in the expression of the STE20-type kinases STK25 and MST4, which are closely related to MST3, was observed in the livers from *Mst3* ASO-treated mice (Additional File 3: Supplementary Figure S1*B*).

We first assessed the total and maximum volume, as well as the number of visible HCC tumors detected during gross inspection of the liver at study termination. Strikingly, we found that all mice developed numerous macroscopic nodules of variable sizes on their liver surface, with no differences between the groups dosed with *Mst3* ASO vs. a non-targeting control ASO (Fig. 1B, C). In line with these macroscopic observations, we concluded that MST3 deficiency had no discernible impact on tumor area quantified in the H&E-stained liver sections, nor on the extent of hepatic immunolabeling for well-established markers of HCC development and progression—alpha-fetoprotein (AFP), Yes-associated protein (YAP), epithelial cell adhesion molecule (EpCAM), and glucose-regulated protein 78 (GRP78) (Fig. 1D–F; Additional File 3: Supplementary Figure S2).

### ASO-mediated knockdown of MST3 protects against hepatic steatotoxicity in a mouse model of MASH-associated HCC

Next, we examined whether the similar liver tumor burden described in *Mst3* ASO-treated and control mice was associated with a comparable degree of MASH severity. Since the number and size of HCC nodules were unaffected by the duration of ASO administration, we focused these investigations on the cohorts that received ASOs for the longest period—30 weeks.

**Fig. 2:**
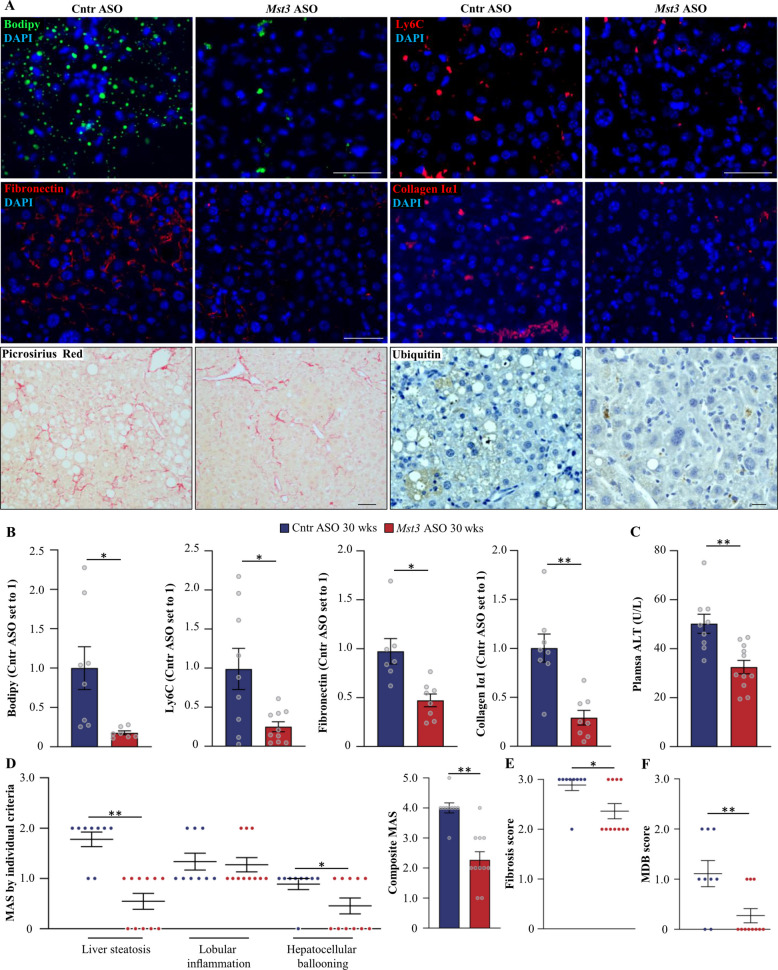
ASO-mediated knockdown of MST3 suppresses liver steatosis, inflammation, and fibrosis in a mouse model of MASH-HCC. **A** Representative images of liver sections stained with Bodipy 493/503 (green) or Picrosirius Red (red), or processed for immunofluorescence/immunohistochemistry with anti-Ly6C (red), anti-fibronectin (red), anti-collagen Iα1 (red), or anti-ubiquitin (brown) antibodies. In immunofluorescence images, nuclei stained with DAPI (blue). In immunohistochemical images for ubiquitin, counterstaining with hematoxylin. Scale bar: 100 μm (Bodipy 493/503, Ly6C, fibronectin, collagen Iα1) or 20 μm (Picrosirius Red, ubiquitin). **B** Quantification of fluorescence-positive area. **C** Measurement of plasma ALT concentrations. **D** Assessment of composite MAS and its individual components (steatosis, lobular inflammation, and hepatocellular ballooning) in H&E-stained liver sections. Scoring was performed by an independent histopathologist based on three semiquantitative components: liver steatosis (0–3), lobular inflammation (0–3), and hepatocellular ballooning (0–2). **E** Quantification of fibrosis score in Picrosirius Red-stained liver sections. (0–4). **F** Scoring of MDBs in H&E-stained liver sections conducted according to the Goodman classification (0–3) [34]. Liver samples were collected from mice treated with *Mst3* ASO or control ASO for 30 weeks. Data are mean ± SEM from 8 to 11 mice per group. Cntr, control; wks, weeks. *p < 0.05, **p < 0.01

Interestingly, despite equivalent HCC tumor load, MST3-deficient mice exhibited marked suppression of key pathological features of diet-induced MASH compared with controls. Specifically, MST3 silencing reduced hepatic steatosis by more than 80%, as evidenced by diminished labeling of liver sections with the lipophilic dye Bodipy 493/503 (Fig. 2A, B), without any alterations in glycogen content (Additional File 3: Supplementary Figure S3). This metabolic improvement was accompanied by subset-specific changes in the hepatic immune composition. Flow cytometry assessment of liver immune populations revealed no statistically significant differences between groups in the total number of liver macrophages (CD45^+^CD11b^+^F4/80^+^), embryonically derived Kupffer cells (CD45^+^CD11b^+^Clec4F^+^Tim4^+^), or monocyte-derived Kupffer cells (CD45^+^CD11b^+^Clec4F^+^Tim4^−^) (Additional File 3: Supplementary Figure S4*A-B*). Quantification of these populations was characterized by substantial inter-individual variability, which may reflect the pronounced phenotypic heterogeneity of hepatic macrophages in advanced MASH [38, 39]. In contrast, although some variability was observed, pro-inflammatory Ly6C^high^ monocytes were consistently reduced in *Mst3* ASO-treated mice compared with controls, as demonstrated by both flow cytometry and immunofluorescence analyses of liver sections (Additional File 3: Supplementary Figure S4*C;* Fig. 2A, B). Given that Ly6C^high^ monocytes represent a major source of inflammatory cytokines and precursors of inflammatory macrophages in MASH [40], their reduction may suggest an overall attenuation of hepatic inflammatory activity in MST3-deficient mice.

Consistent with preceding findings, mice dosed with *Mst3*-targeting ASO were protected from liver fibrosis, as demonstrated by immunofluorescence analysis for fibronectin and collagen Iα1, as well as Picrosirius Red staining, which detects collagen I and III (Fig. 2A, B). Plasma concentrations of ALT, a key clinical biomarker of MASH, were also lower in MST3-deficient vs. control mice (Fig. 2C). In line with these results, ASO-mediated inhibition of MST3 decreased the MAS (comprising hepatic steatosis, lobular inflammation, and hepatocellular ballooning scores) and the fibrosis stage, both evaluated by histopathological analysis of liver sections using the Kleiner/Brunt criteria adapted for use in rodents [32, 33] (Fig. 2D, E). Furthermore, Mallory-Denk bodies (MDBs)—a hallmark of MASH activity recently incorporated into the expanded MAS criteria [34]—were more abundant in livers from control mice compared with MST3-deficient mice, as shown by histological scoring in H&E-stained liver sections according to the Goodman classification [34] (Fig. 2F) as well as by immunohistochemical and immunofluorescence assessment for the MDB constituents ubiquitin and p62, respectively (Fig. 2A*;* Additional File 3: Supplementary Figure S5).

### MST3 silencing attenuates hepatocellular oxidative and ER stress while stimulating mitochondrial and lysosomal marker expression

**Fig. 3:**
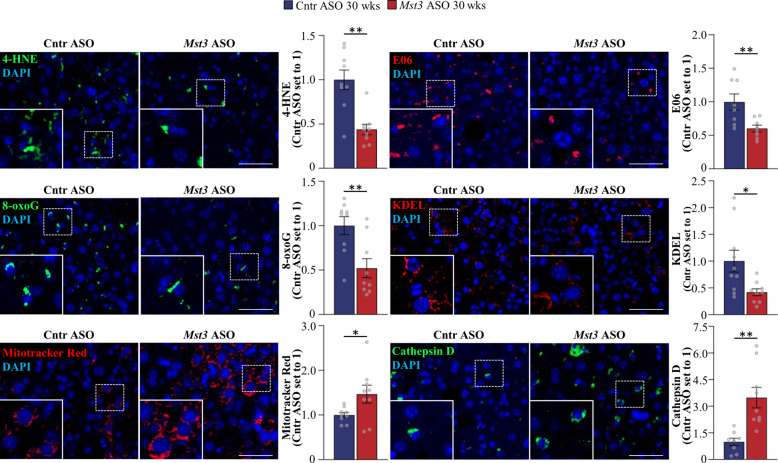
MST3 silencing reduces hepatocellular oxidative and ER stress while enhancing mitochondrial and lysosomal marker expression. Representative images of liver sections stained with Mitotracker Red (red) or processed for immunofluorescence with anti-4-HNE (green), anti-E06 (red), anti-8-oxoG (green), anti-KDEL (red), or anti-cathepsin D (green) antibodies; nuclei stained with DAPI (blue). Scale bar: 100 μm. Quantification of fluorescence-positive area. Liver samples were collected from mice treated with *Mst3* ASO or control ASO for 30 weeks. Data are mean ± SEM from 8 to 9 mice per group. Cntr, control; wks, weeks. *p < 0.05, **p < 0.01

Lipotoxicity-induced oxidative and ER stress, along with dysregulated autophagy, play pivotal roles in the development of both MASH and HCC by driving hepatic inflammation, hepatocyte dysfunction, and ultimately, liver injury [12]. To this end, we investigated the markers of oxidative and ER stress in the livers sections from mice treated with *Mst3*-targeting or control ASO for 30 weeks. We detected a significant reduction in hepatic oxidative damage in MST3-deficient mice, as evidenced by diminished area stained for 4-HNE (an end product of peroxidation of membrane *N*−6-polyunsaturated fatty acids), E06 (a marker of oxidized phospholipids), and 8-oxoG (the most common oxidation product of DNA) (Fig. 3). In addition, we found a suppression of hepatic ER stress in mice receiving *Mst3* ASO vs. control ASO, as indicated by lower immunolabeling for KDEL, a signal motif for ER retrieval (Fig. 3). Notably, MST3 knockdown did not affect autophagic activity, as reflected by comparable LC3-II to LC3-I ratios in the livers from both groups (Additional File 3: Supplementary Figure S6).

**Fig. 4:**
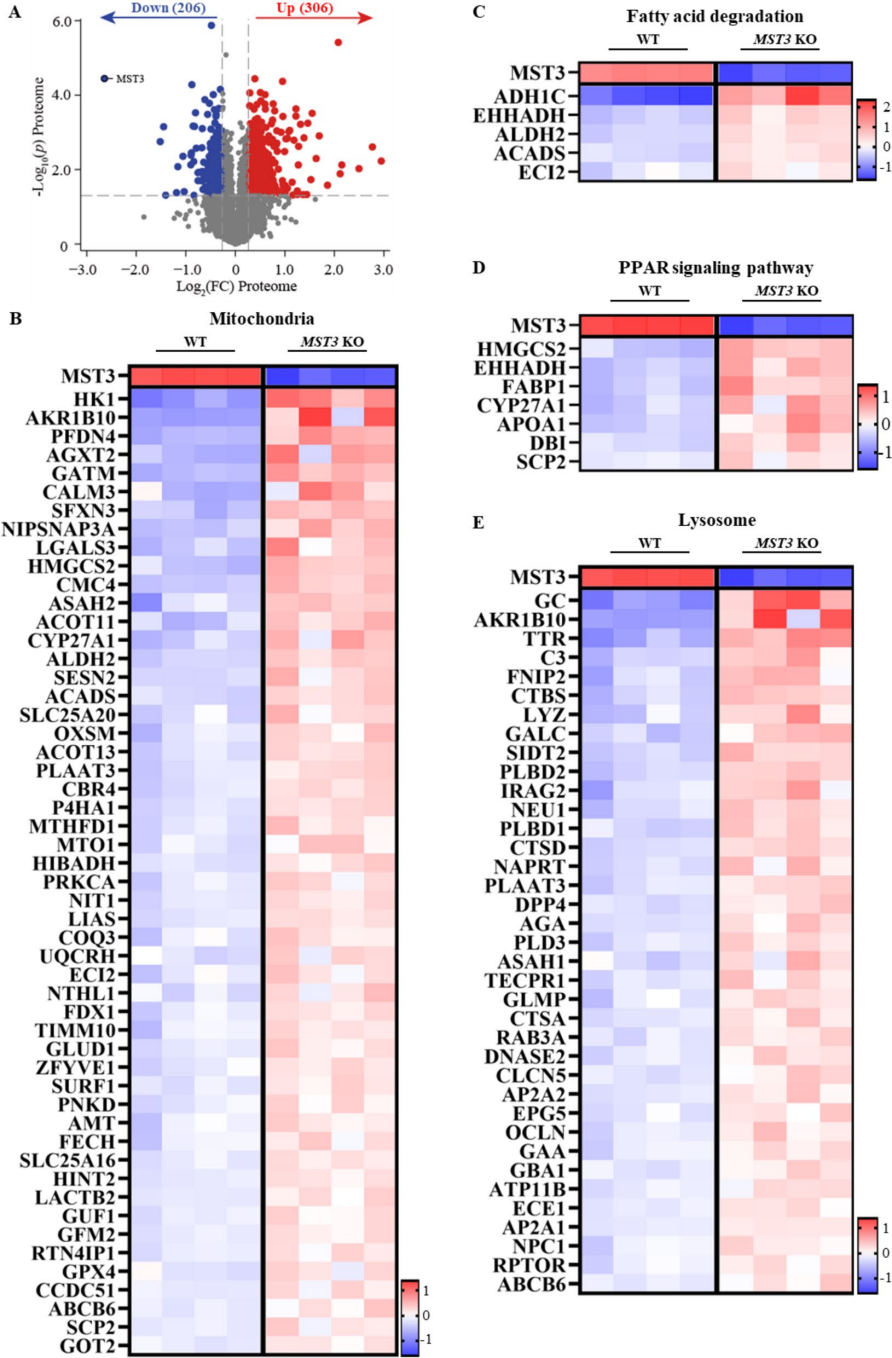
Comparison of whole-cell proteomic profiles between *MST3* knockout and wild-type Huh7 cells. **A** Volcano plot of differentially represented proteins. **B**–**E** Heat maps of the enrichment analyses of significantly altered mitochondrial and lysosomal proteins (**B**, **E**) and proteins involved in fatty acid degradation (**C**) and PPAR signaling pathways (**D**). The scaled abundance of four different clones per genotype is shown. The protein names are denoted on the left. KO, knockout; WT, wild-type

To further investigate the hepatocellular mechanism of action of MST3, we performed relative quantification of the whole-cell proteome in *MST3* knockout vs. wild-type Huh7 cells (a well-differentiated human HCC cell line). A total of 7582 proteins were identified, among which 306 were upregulated and 206 were downregulated (*t*-test, |log₂FC|≥ 0.27) (Fig. 4A). Enrichment analysis revealed increased abundance of numerous mitochondrial proteins in MST3-deficient cells, including translation factors (GFM2, GUF1, MTO1), electron transport chain components (COQ3, UQCRH, SURF1), proteins mediating membrane insertion and metabolite transport (TIMM10, SLC25A16), β-oxidation and cholesterol clearance enzymes (ACADS, CYP27A1, HMGCS2, SLC25A20, ACOT11, ACOT13, ECI2), and antioxidant defense markers (GPX4, SESN2, FDX1) (Fig. 4B). In line with the upregulation of the mitochondrial marker expression, we also found elevated representation of proteins involved in fatty acid degradation and PPAR signaling pathways in *MST3* knockout cells compared with wild-type cells (Fig. 4C, D). In parallel, depletion of MST3 induced the expression of lysosomal markers, including proteases (CTSD, CTSA), glycosidases (GBA1, GAA, NEU1), lipid-processing and cholesterol transport proteins (ASAH1, GALC, PLBD1, PLBD2, NPC1), and membrane trafficking and transport-associated proteins (CLCN5, ATP11B, ABCB6, AP2A1, AP2A2, RAB3A), which is indicative of augmented degradative and lipid clearance capacity (Fig. 4E). Consistent with proteomic analysis in Huh7 cells, we detected enhanced staining for Mitotracker Red and increased immunolabelling for the main lysosomal protease cathepsin D (CTSD) in the livers from *Mst3* ASO-treated mice compared with controls (Fig. 3). Together, the coordinated activation of mitochondrial and lysosomal pathways is expected to promote fatty acid degradation and catabolic clearance, in line with the reduced hepatic lipid accumulation observed in MST3-deficient mice. However, metabolic regulation in Huh7 cells used for proteomic studies may not fully reflect that of non-transformed hepatocytes, a limitation to consider when interpreting the results.

Given the absence of coordinated changes in tumorigenesis-related pathways by enrichment analysis, we performed a targeted, literature-guided evaluation of proteins with established roles in HCC, comparing their abundances in *MST3* knockout and wild-type Huh7 cells. Specifically, we focused on the mitogen-activated protein kinase (MAPK; ERK1/2, JNK1/2, and p38α/$\gamma$), Janus kinase (JAK)/signal transducer and activator of transcription (STAT), ErbB, and Hippo pathways, which represent key signaling components controlling proliferation, differentiation, and migration in human HCC [41–46]. Applying this targeted approach, we found that only two proteins within the ErbB network were differentially expressed—EGFR was upregulated, while GRB14 was downregulated in MST3-deficient cells—and no coordinated alterations were detected across the examined pathways (Additional File 3: Supplementary Figure S7). We acknowledge that the assessment was limited to total protein amounts, whereas the activity of these signaling pathways is additionally controlled by post-translational modifications, particularly phosphorylation.

### *Mst3* ASO treatment decreases adiposity and improves glucose and insulin homeostasis in a MASH-driven HCC mouse model

**Fig. 5:**
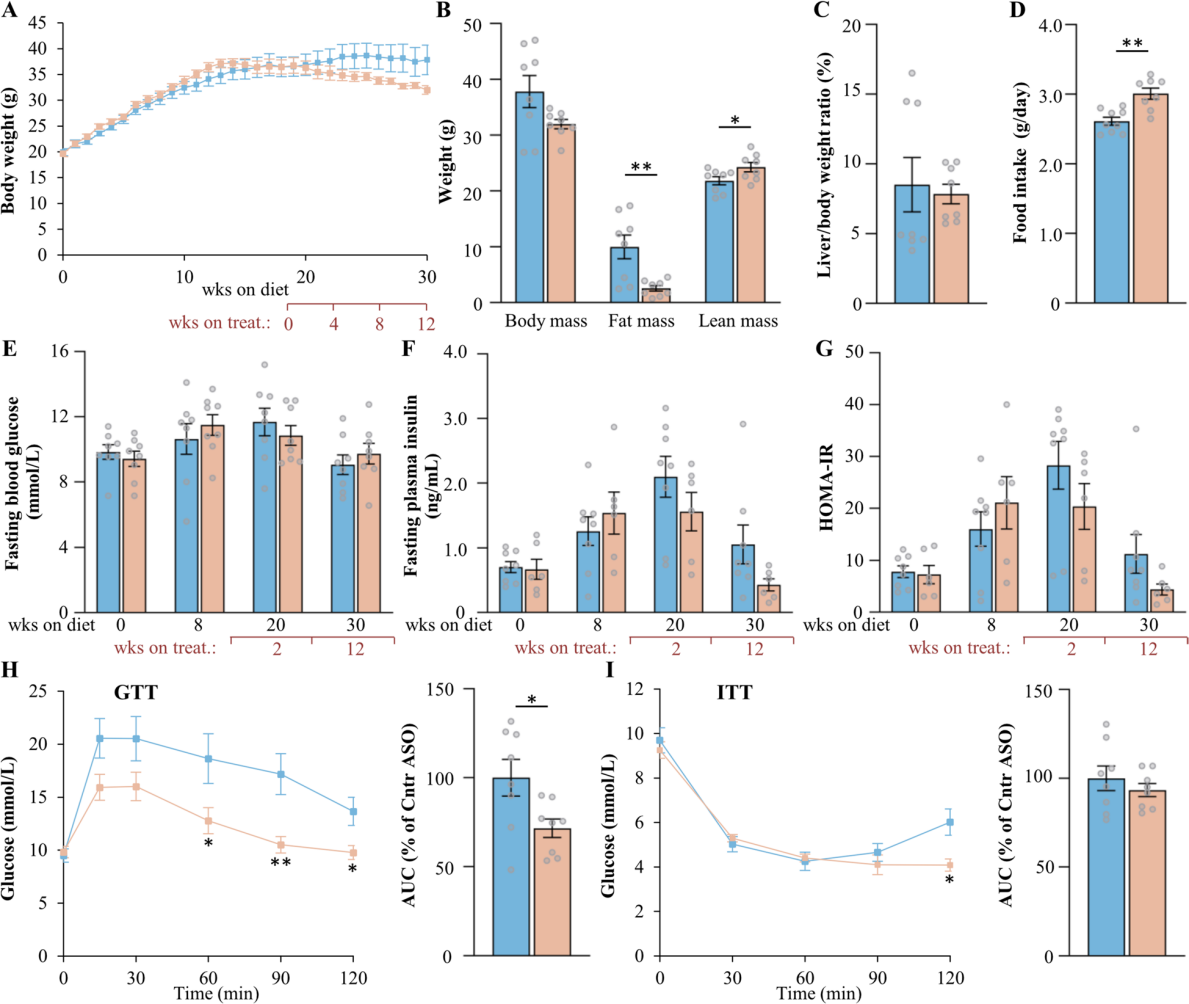
Assessment of body weight and composition, and glucose and insulin homeostasis, in mice with MASH-HCC following 12 weeks of treatment with *Mst3* ASO or control ASO. **A** Body weight curves. **B** Total, fat, and lean body mass determined by BCA. **C** Liver-to-body weight ratio. **D** Accumulated food consumption monitored per day. **E**, **F** Fasting circulating levels of glucose (**E**) and insulin (**F**). **G** HOMA-IR calculated as (fasting glucose [mmol/L] × fasting insulin [ng/mL])/22.5. **H**, **I** Intraperitoneal GTT (**H**) and ITT (**I**) performed after 9 and 10 weeks of ASO treatment, respectively. The area under the glucose curve (AUC) for each test is shown. Data are mean ± SEM from 7 to 8 mice per group. Cntr, control; wks, weeks. *p < 0.05, **p < 0.01

**Fig. 6:**
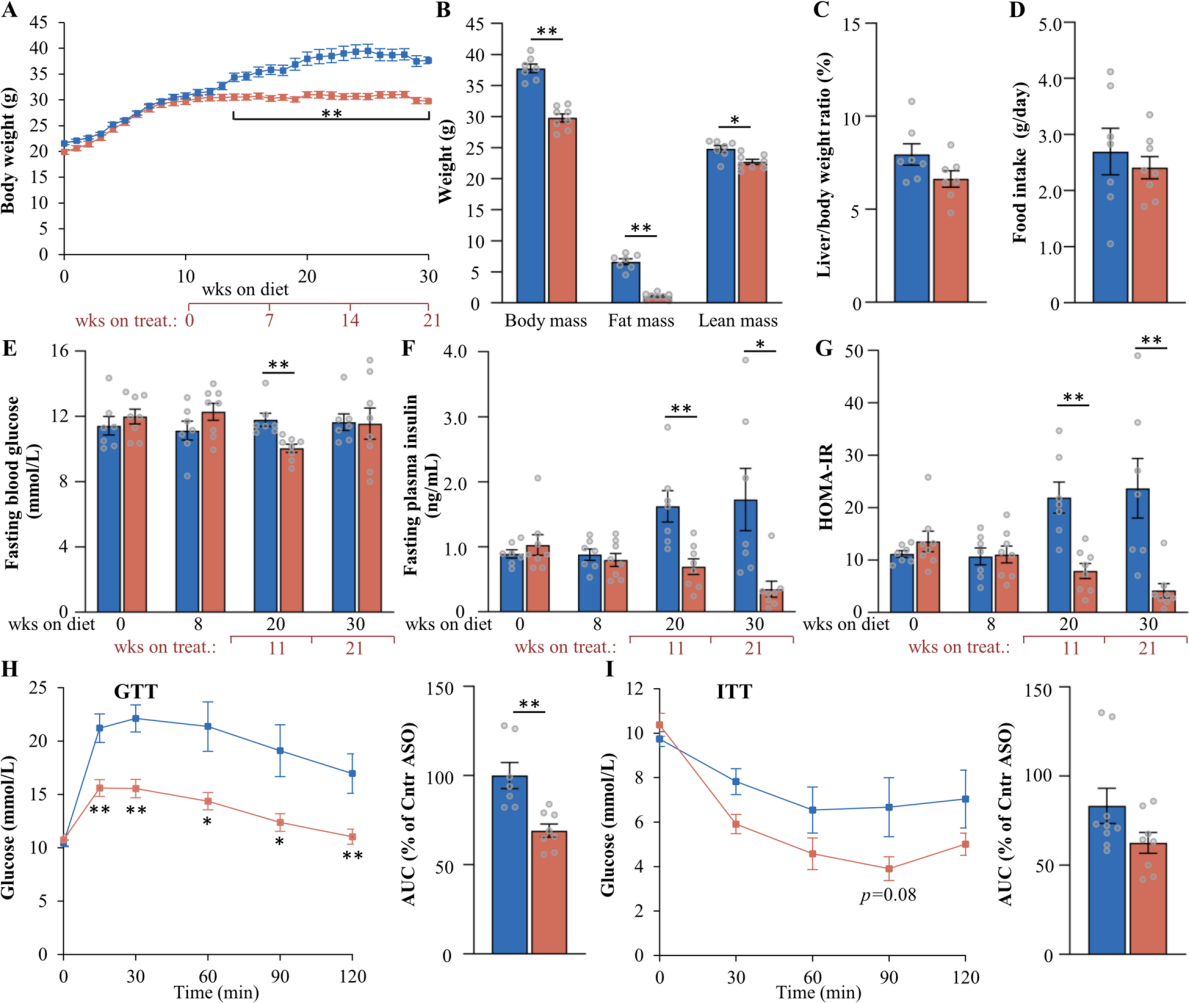
Assessment of body weight and composition, and glucose and insulin homeostasis, in mice with MASH-HCC following 21 weeks of treatment with *Mst3* ASO or control ASO. **A** Body weight curves. **B** Total, fat, and lean body mass determined by BCA. **C** Liver-to-body weight ratio. **D** Accumulated food consumption monitored per day. **E**, **F** Fasting circulating levels of glucose (**E**) and insulin (**F**). **G** HOMA-IR calculated as (fasting glucose [mmol/L] × fasting insulin [ng/mL])/22.5. **H**, **I** Intraperitoneal GTT (**H**) and ITT (**I**) performed after 18 and 19 weeks of ASO treatment, respectively. AUC for each test is shown. Data are mean ± SEM from 7 to 8 mice per group. Cntr, control; wks, weeks. *p < 0.05, **p < 0.01

**Fig. 7:**
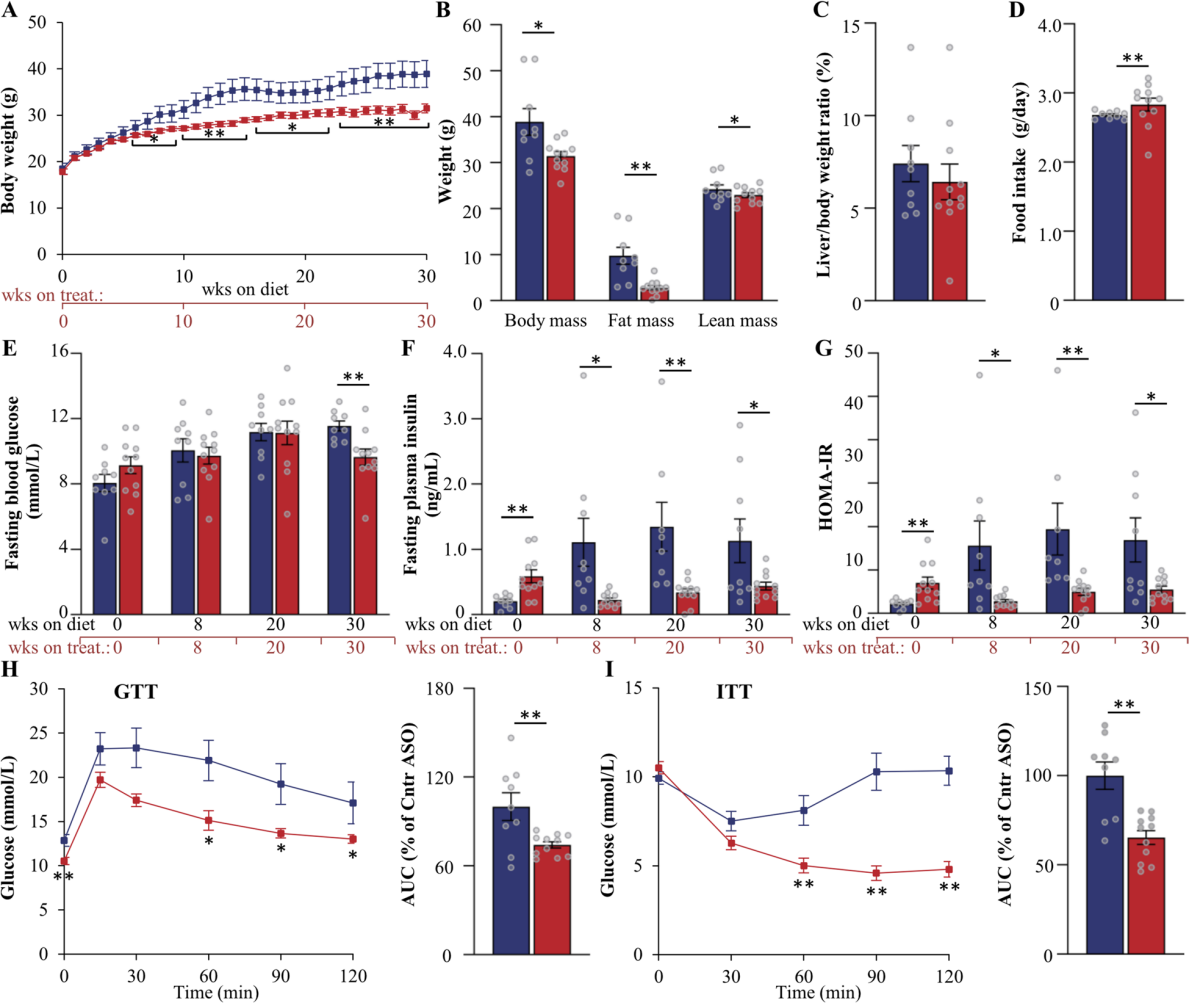
Assessment of body weight and composition, and glucose and insulin homeostasis, in mice with MASH-HCC following 30 weeks of treatment with *Mst3* ASO or control ASO. **A** Body weight curves. **B** Total, fat, and lean body mass determined by BCA. **C** Liver-to-body weight ratio. **D** Accumulated food consumption monitored per day. **E**, **F** Fasting circulating levels of glucose (**E**) and insulin (**F**). **G** HOMA-IR calculated as (fasting glucose [mmol/L] × fasting insulin [ng/mL])/22.5. **H**, **I**) Intraperitoneal GTT (**H**) and ITT (**I**) performed after 27 and 28 weeks of ASO treatment, respectively. AUC for each test is shown. Data are mean ± SEM from 7 to 8 mice per group. Cntr, control; wks, weeks. *p < 0.05, **p < 0.01

Finally, we investigated the impact of ASO administration on measures of whole-body metabolic physiology. We found that treatment with *Mst3-*targeting ASO for 12, 21, or 30 weeks significantly decreased fat mass in obese mice (Figs. 5B, 6B, and 7B). MST3 deficiency did not result in consistent changes in fasting blood glucose levels measured at several time points during the study (Figs. 5E, 6E, and 7E). However, plasma insulin concentrations and the homeostasis model assessment score of insulin resistance (HOMA-IR) were lower in the cohorts of mice that received *Mst3* ASO for 21 or 30 weeks compared with their respective controls (Figs. 5F, G, 6F, G, and 7F, G). Interestingly, suppression of MST3 abundance improved glucose tolerance, as assessed by GTT, in all three treatment groups (Figs. 5H, 6H, and 7H). In contrast, enhanced insulin sensitivity measured by ITT was only detected in the cohort dosed with *Mst3* ASO for 30 weeks (Figs. 5I, 6I, and 7I). Of note, we observed a slight increase in food intake and lean mass in mice treated with *Mst3* ASO during the 12-week period, and a modest reduction in lean mass in mice dosed for 21 or 30 weeks, when compared with controls (Figs. 5B, D, 6B, D, and 7B, D). These findings were considered incidental, with no clear underlying explanation or apparent physiological relevance.

## Discussion

Here, we provide the first in vivo evidence that treatment with *Mst3*-targeting ASO does not alter hepatocarcinogenesis in a mouse model of MASH-associated HCC, in which disease was induced by a DEN injection combined with Western-style diet feeding. This conclusion is supported by comparable numbers and sizes of visible liver tumors, as well as similar histological indices of HCC, in *Mst3*-deficient and control groups. Notably, the lack of an effect on tumor development occurred despite potent reductions in hepatic *Mst3* mRNA and protein levels and a pronounced suppression of MASH severity observed following *Mst3* ASO dosing.

The results of the present in vivo investigations—showing no phenotypic impact of MST3 knockdown on HCC onset or progression in mice—were unexpected in light of our recent in vitro studies, which indicate a potential oncogenic function of MST3 in hepatocarcinogenesis [28]. Specifically, we previously reported that silencing of MST3 via small interfering RNA (siRNA) markedly suppresses tumorigenic features in cultured human hepatocyte-derived cell lines—including well-differentiated and poorly differentiated HCC cells, as well as hepatoblastoma cells—by inhibiting proliferation, migration, invasion, and epithelial-mesenchymal transition (EMT) capabilities [28]. We consider it unlikely that the discrepancy between in vivo and in vitro findings is attributable to species-specific differences in gene function, as MST3 is highly conserved in mice and humans, with an amino acid sequence identity of 97.1%. However, it is important to emphasize that in vitro experiments using immortalized human hepatocyte cell lines do not fully recapitulate the complex liver microenvironment—comprising parenchymal cells, non-parenchymal cells, and the extracellular matrix—which represents a pivotal determinant in the development of MASH-associated HCC [5]. Moreover, we speculate that STE20-type kinases, which are closely related to MST3 and exhibit expression patterns and hepatocellular activities that overlap with those of MST3 [14], may compensate for the absence of MST3 in mice but not in cultured cells. Finally, discrepancies in the pharmacokinetics and bioavailability of the agents used for gene silencing in the animal model vs. the cell culture system may have affected their efficacy. Of note, these potential explanatory mechanisms are hypothetical, and the reasons for the divergence between in vitro and in vivo findings remain unknown.

MST3 shares close homology with STK25, as both proteins belong to the GCKIII subgroup of the STE20 kinase family and contain a highly conserved catalytic domain, with approximately 90% amino acid sequence identity in both mice and humans [14]. In line with the phenotypic effects of MST3 deficiency described in this report and our previous work [26], depletion of STK25 has been shown to repress ectopic lipid deposition in human hepatocytes and to confer strong protection against diet-induced MASH in mice [15, 20–22, 24, 25]. Furthermore, similar to MST3 [28], in vitro silencing of STK25 suppresses tumorigenic capacity in human HCC cell lines [29, 47]. Importantly, in contrast to the comparable tumor burden observed in mice treated with *Mst3*-targeting or control ASO in this study, both genetic ablation and pharmacologic inhibition of STK25—achieved via systemic or hepatocyte-specific ASO delivery—efficiently mitigate the initiation and exacerbation of hepatocarcinogenesis in a mouse model of MASH-associated HCC [29, 30]. Interestingly, our recent research demonstrated an equal reduction in MASH severity in obese mice with the combined deficiency of MST3 and STK25, compared with mice lacking only one of the kinases [48]. These findings are consistent with our earlier in vitro investigations in immortalized human hepatocytes, where individual knockdown of MST3 or STK25 results in a similar decrease in intracellular lipid content and oxidative/ER stress, without any additive or synergistic effects detected upon simultaneous silencing of both kinases [21]. Taken together, while the molecular hierarchy and functional relationship between MST3 and STK25 remain to be established, current evidence suggests that these two kinases may operate within the same signaling axis and/or engage certain common pathways, with overlapping—but not identical—roles in regulating liver homeostasis and contributing to its dysfunction. This interpretation is consistent with previous research that has revealed multiple shared interaction partners and phosphorylation targets for MST3 and STK25 [21].

In this study, we report reduced body weight and fat mass, along with improved whole-body glucose and insulin homeostasis, in Western-style diet-fed mice treated with *Mst3*-targeting vs. control ASO. Notably, these effects were most pronounced in cohorts dosed for extended durations (i.e., 21 or 30 weeks), indicating that sustained reduction in MST3 activity may be necessary to elicit metabolic benefits. Consistent with this interpretation, our earlier observations in high-fat-fed mice treated with *Mst3* ASO for a shorter period of 10 weeks did not reveal significant alterations in body weight, fasting blood glucose or insulin levels, or glucose tolerance or insulin sensitivity, as assessed by GTT and ITT, respectively [26]. Interestingly, previous research demonstrated that obese *Mst3* gene trap mice—which exhibit a marked, though incomplete, decrease in *Mst3* expression—present with no changes in body weight, fat mass, or systemic glucose tolerance; however, similar to the findings in this study, they display increased insulin sensitivity [23]. The reasons for these somewhat conflicting results may include potential developmental impacts that manifest in response to genetic knockdown of MST3 but are not replicated by pharmacological inactivation of the target; differences in the genotypic backgrounds of the mice used (mixed 129 Sv/C57BL/6 J in *Mst3* gene trap mice vs. pure C57BL/6 J in *Mst3* ASO-treated mice); variations in the diets employed (40–45 kcal% fat and 35–40 kcal% sugars, with differences in the lipid and carbohydrate sources); and/or disparities in the extent of *Mst3* inhibition achieved in the two models. It should be emphasized that the proposed explanations remain speculative, as resolving the phenotypic differences between the two models would require additional dedicated in vivo experiments, which were not performed in the current study, representing a limitation of the work.

The prevailing view is that MASH is required for the development of HCC in obesity. Nevertheless, we show here that pharmacological MST3 inhibition in the selected mouse model improved MASH without altering HCC, indicating that the effects of MST3 antagonism on MASH and HCC can be dissociated. Interestingly, previous studies have demonstrated that obesity-associated oxidative stress can independently affect MASH and HCC [44]. In this context, the oxidative hepatic environment in high-fat diet-fed mice inactivates STAT1 and STAT3 phosphatase, thereby enhancing STAT1 and STAT3 signaling, with selective attenuation of STAT1 activity preventing MASH and fibrosis but not HCC, whereas correction of STAT3 activity suppressing HCC without any impact on MASH or fibrosis [44]. Of note, consistent with lower hepatic oxidative stress, we observed markedly diminished STAT3 signaling in whole liver lysates from *Mst3* ASO-treated mice compared with controls (Additional File 3: Supplementary Figure S8). However, under the experimental conditions applied in this study, the reduction in hepatic STAT3 signaling in MST3-deficient mice was clearly not sufficient to repress HCC tumor burden.

From a translational standpoint, MST3 inhibition could potentially be beneficial in patients with early-stage MASLD, characterized by hepatic steatosis and low-grade metabolic inflammation in the absence of advanced fibrosis, a population with a relatively low risk of HCC. Clinically, such patients may be defined by imaging-based evidence of hepatic steatosis together with normal or only mildly elevated liver stiffness on transient elastography (< 8 kPa), and/or by histological features consistent with MASLD and a low fibrosis stage (F0–F1). In this context, targeting MST3 could represent a potential disease-modifying strategy aimed at halting or delaying progression toward fibrotic MASH, in line with evidence that aggravation of fibrosis per se increases HCC risk in patients with MASLD [49, 50]. Importantly, these translational implications should be interpreted with caution, and additional nonclinical studies in complementary experimental models, as well as clinical trials in patients with MASLD/MASH, are required to establish the efficacy and safety of MST3 inhibition.

A limitation of this investigation is the use of a single mouse model, where hepatocarcinogenesis was triggered in a MASH-like context by administration of DEN—a chemical procarcinogen that alkylates DNA and drives oxidative stress through the generation of reactive oxygen species (ROS) [51]—in combination with long-term feeding of a Western-style diet rich in fat, cholesterol, and fructose to induce hepatic steatosis, inflammation, cellular injury, and fibrosis [52]. The diet was chosen based on a recent unbiased ranking of murine dietary regimens according to their proximity to human MASLD that identified the Western-style diet employed here as one of the most balanced in terms of metabolic, histologic, and transcriptomic similarities to the human disease [52]. DEN exposure was included because, compared with diet-only approaches that require prolonged feeding to induce HCC, combinations of DEN and dietary challenge provide accelerated tumor initiation while preserving diet-driven MASH pathology [53, 54]. While this model is valuable for replicating key features of human disease, it may not fully capture the slow, multifactorial nature of tumorigenesis or accurately mirror the complex tumor microenvironment characteristic for MASH-HCC in humans. Moreover, it represents a particularly aggressive disease setting, in which exposure to an exogenous carcinogen may mask more subtle tumor-modifying effects of MST3 inhibition. Future studies employing complementary models—such as genetically engineered mice that develop MASH-related HCC without exogenous carcinogens, immunologically humanized mice, or human liver organoids—will be required to further assess the generalizability and translational relevance of the findings.

## Conclusions

In summary, while MST3 is dispensable for hepatocarcinogenesis, its antagonism prevents diet-induced metabolic dysfunction and attenuates MASH severity in the selected mouse model of MASH-HCC. Although the precise molecular mechanisms underlying MST3’s effects remain incompletely understood, our findings challenge the prevailing assumption that targeting MASH driver genes alone is sufficient to mitigate HCC and suggest that an effective therapeutic strategy may require a multi-pronged approach—such as combining metabolism-targeting interventions with distinct anti-tumorigenic agents—to comprehensively address the full clinical spectrum of MASH-associated HCC.

## Supplementary Information

Additional file 1: Supplementary Tables S1-S2. Supplementary Table S1. List of antibodies. Supplementary Table S2. List of sgRNAs used for CRISPR/Cas9 editing.

Additional file 2: Supplementary Methods. CRISPR/Cas9-Mediated *MST3* Knockout in Huh7 Cells and Whole-Cell Proteomics.

Additional file 3: Supplementary Figures S1-S8. Figure S1. Expression of MST3 and related protein kinases in the livers from mice with MASH-HCC. Figure S2. Analysis of hepatic EpCAM and GRP78 abundance in mice with MASH-HCC. Figure S3. Analysis of hepatic glycogen levels in mice with MASH-HCC. Figure S4. Flow cytometric assessment of hepatic immune composition in mice with MASH-HCC. Figure S5. Analysis of hepatic p62 abundance in mice with MASH-HCC. Figure S6. Analysis of relative LC3-II to LC3-I ratio in the livers from mice with MASH-HCC. Figure S7. Comparison of whole-cell proteomic profiles between *MST3* knockout and wild-type Huh7 cells. Figure S8. Analysis of relative phospho-STAT3 to STAT3 ratio in the livers from mice with MASH-HCC.

Additional file 4: Images of the original, uncropped Western blots.

## Data Availability

The data that support the findings of this study are available on request from the corresponding author.

## Abbreviations

AFP: Alpha-fetoprotein
ALT: Alanine aminotransferase
ASO: Antisense oligonucleotide
BCA: Body composition analysis
CTSD: Cathepsin D
DEN: Diethylnitrosamine
EMT: Epithelial-mesenchymal transition
EpCAM: Epithelial cell adhesion molecule
ER: Endoplasmic reticulum
GTT: Glucose tolerance test
GRP78: Glucose-regulated protein 78
H&E: Hematoxylin and eosin
HCC: Hepatocellular carcinoma
HOMA-IR: Homeostasis model assessment of insulin resistance
ITT: Insulin tolerance test
JAK: Janus kinase
MAPK: Mitogen-activated protein kinase
MASLD: Metabolic dysfunction-associated steatotic liver disease
MAS: MASLD activity score
MASH: Metabolic dysfunction-associated steatohepatitis
MDBs: Mallory-Denk bodies
NIH: National Institutes of Health
STAT: Signal transducer and activator of transcription
ROS: Reactive oxygen species
sgRNAs: Single-guide RNAs
TD-NMR: Time-domain nuclear magnetic resonance
YAP: Yes-associated protein
