# Supplementary material for: Targeting STE20-type kinase MST3 improves metabolic dysfunction-associated steatohepatitis without affecting hepatocellular carcinoma development in mice
Source: BMC Med. 2026 Mar 24;24:214. doi: 10.1186/s12916-026-04812-0 (PMC13063862; doi:10.1186/s12916-026-04812-0)
Supplement: Supplementary file 1 — Additional file 1: Supplementary Tables S1-S2. Supplementary Table S1. List of antibodies. Supplementary Table S2. List of sgRNAs used for CRISPR/Cas9 editing. [file 12916_2026_4812_MOESM1_ESM.pdf]

Supplementary Table S1. List of antibodies used for immunofluorescence/immunohistochemistry and Western blot analysis

Type	Antibody name and catalog number	Working dilution	Company
Primary antibody	anti-MST3 (#3723)	1:1000	Cell Signaling Technology (Boston, MA)
	anti-EpCAM (ab213500)	1:100	Abcam (Cambridge, UK)
	anti-AFP (ab46799)	1:200	Abcam
	anti-GRP78 (sc-166490)	1:250	Santa Cruz Biotechnology (Santa Cruz, CA)
	anti-YAP (ab205270)	1:500	Abcam
	anti-vinculin (sc-25336)	1:500	Santa Cruz Biotechnology
	anti-Gr1 (Ly6C) (ab15627)	1:300	Abcam
	anti-collagen Ia1 (NBP1-30054)	1:100	Novus Biologicals (Centennial, CO)
	anti-fibronectin (F3648)	1:300	Sigma-Aldrich (St. Louis, MO)
	anti-ubiquitin (ab7780)	1:200	Abcam
	anti-SQSTM1/p62 (ab91526)	1:300	Abcam
	anti-4-HNE (ab46545)	1:500	Abcam
	anti-E06 (330001S)	1:100	Avanti Polar Lipids, Inc (Alabaster, AL)
	anti-8-oxoG (ab62623)	1:500	Abcam
	anti-KDEL (ab176333)	1:500	Abcam
	anti-Cathepsin D (ab75852)	1:100	Abcam
	anti-LC3 (#2775)	1:1000	Cell Signaling Technology
	anti-STAT3 (#4904)	1:1000	Cell Signaling Technology
	anti-p-STAT3 (#9131)	1:500	Cell Signaling Technology
Secondary antibody	Alexa Fluor-488-labeled anti-mouse IgG (A21202)	1:500	Invitrogen (Waltman, MA)
	Alexa Fluor-594-labeled anti-rat IgG (A11007)	1:500	Invitrogen
	Alexa Fluor-594-labeled anti-rabbit IgG (A21207)	1:500	Invitrogen
	Alexa Fluor-594-labeled anti-mouse IgG (A11005)	1:500	Invitrogen
	anti-rabbit IgG (#7074)	1:1000	Cell Signaling Technology
	anti-mouse IgG (#7076)	1:1000	Cell Signaling Technology
	Biotinylated-labeled anti-rabbit IgG (E0432)	1:300	Dako (Carpenteria, CA)
Fluorophore-conjugated antibody	BV711 anti-CD45 (563709)	1:400	BD Bioscience (Franklin Lakes, NJ)
	BV510 anti-F4/80 (123135)	1:200	Biolegend (San Diego, CA)
	BV786 anti-CD11b (740861)	1:200	BD Bioscience
	Alexa647 anti-Clec4F (156803)	1:100	Biolegend
	Alexa488 anti-Tim4 (53-5866-82)	1:200	Invitrogen
	APC anti CD11b (101212)	1:400	Biolegend

Supplementary Table S2. List of sgRNAs used for CRISPR/Cas9 editing

Name	Position in genome	Strand	Sequence
MST3-ERegion2a	58568	+	TGACAATCGGACTCAGAAAG
MST3-ERegion2b	58694	+	ATATTATGGATCCTATCTGA
MST3-ERegion4	102639	+	ATATTAAGAGAAATACTGAA
