# Supplementary material for: Targeting STE20-type kinase MST3 improves metabolic dysfunction-associated steatohepatitis without affecting hepatocellular carcinoma development in mice
Source: BMC Med. 2026 Mar 24;24:214. doi: 10.1186/s12916-026-04812-0 (PMC13063862; doi:10.1186/s12916-026-04812-0)
Supplement: Supplementary file 2 — Additional file 2: Supplementary Methods. CRISPR/Cas9-Mediated MST3 Knockout in Huh7 Cells and Whole-Cell Proteomics. [file 12916_2026_4812_MOESM2_ESM.pdf]

SUPPLEMENTARY METHODS

CRISPR/Cas9-Mediated MST3 Knockout in Huh7 Cells

Three single-guide RNAs (sgRNAs) targeting coding exons of *MST3* (see Additional File 1: Supplementary Table S2) were designed using an online CRISPR design tool, and annealed 20-nt oligonucleotides were cloned into the BsmBI site of the lentiGuide-Puro vector (Addgene, 52963; a gift from Feng Zhang) as previously described. Plasmids were purified using the ZymoPURE Miniprep Kit (Zymo Research, Irvine, CA). For lentiviral production, HEK293T cells were co-transfected with LentiCas9-Blast (Addgene, 52962) or lentiGuide-Puro-sgRNA, together with pCMV-dR8.2 (Addgene, 8455) and pCMV-VSV-G (Addgene, 8454), using X-tremeGENE 9 (XTG9-RO; Roche). Viral supernatants were harvested 60 hours post-transfection and filtered. Huh7 cells were transduced with LentiCas9-Blast and selected with 10 µg/mL Blasticidin S HCl (A1113903; Gibco, Paisley, UK) for seven days to establish a stable Cas9-expressing line, confirmed by Western blot using an anti-Cas9 antibody. Cas9-expressing Huh7 cells were subsequently infected with lentiGuide-Puro-sgRNA lentivirus and selected with 3 µg/mL Puromycin (A1113803; Gibco) for seven days, generating a pooled knockout population, with loss of MST3 confirmed by Western blot. Puromycin-resistant cells were subjected to limiting dilution, and single-cell-derived clones were expanded and validated for complete loss of MST3 by Western blot.

MST3 knockout and wild-type Huh7 cells were cultured in Dulbecco's Modified Eagle's Medium (DMEM; GlutaMAX, Gibco) supplemented with 10% (vol/vol) FBS and 1% (vol/vol) penicillin/streptomycin (Gibco), and were routinely confirmed to be mycoplasma-free using the MycoAlert Mycoplasma Detection Kit (Lonza, Basel, Switzerland).

Whole-Cell Proteomics

For proteomic analysis, *MST3* knockout and wild-type cells (n=4 clones/genotype) were lysed using a FastPrep instrument (Matrix D; MP Biomedicals) according to the manufacturer's instructions. Proteins were processed using a modified SP3 protocol. In brief, samples (200 µg) were reduced with 10 mmol/L dithiothreitol (DTT; 30 minutes, 56 °C) and alkylated with 20 mmol/L iodoacetamide (IAA; 30 minutes, room temperature). Proteins were precipitated on Sera-Mag SpeedBeads (Cytiva, Marlborough, MA) by acetonitrile, washed, and dried at room temperature. The beads were resuspended in 50 mmol/L triethylammonium bicarbonate (TEAB), and the proteins were digested by Trypsin/Lys-C mix (Promega,

Madison, WI) for two hours, followed by digestion with trypsin (1:50; Thermo Fisher Scientific) overnight. Beads were removed, peptides were labelled using TMTpro 18-plex isobaric mass tagging reagents (Thermo Fisher Scientific) and combined into a single TMT-set. Desalted peptides were pooled and fractionated into 36 fractions by basic reversed-phase liquid chromatography (bRP-LC, pH 10; Thermo Fisher Scientific). Each fraction was subjected to MS acquisition on an Orbitrap Eclipse Tribrid mass spectrometer equipped with FAIMS Pro interfaced with nLC 1200 liquid chromatography system (all Thermo Fisher Scientific). Peptides were separated on a 40 cm C18 column using a 90-minute gradient, and data was acquired with a SPS MS3 method. Raw data files were processed within Proteome Discoverer (version 3.0; Thermo Fisher Scientific) by matching against SwissProt *Homo sapiens* using Sequest as a search engine. Relative quantification was based on TMT reporter ion intensities. Only unique peptides were considered for the protein quantification (SPS match of 65%). Peptides were filtered for high-confidence identification, and the resulting proteins were retained at medium confidence. Statistical analyses of proteomic data were conducted to identify differentially expressed proteins with Welch's *t*-test performed on log2-transformed data. Proteins with a p-value < 0.05 and fold-change $|\log_2 FC| \geq 0.27$ were considered as differentially represented. Volcano plots and heat maps were generated using GraphPad Prism version 10.0.2 (GraphPad Software, San Diego, CA).
