# Supplementary material for: Targeting STE20-type kinase MST3 improves metabolic dysfunction-associated steatohepatitis without affecting hepatocellular carcinoma development in mice
Source: BMC Med. 2026 Mar 24;24:214. doi: 10.1186/s12916-026-04812-0 (PMC13063862; doi:10.1186/s12916-026-04812-0)
Supplement: Supplementary file 3 — Additional file 3: Supplementary Figures S1-S8. Figure S1. Expression of MST3 and related protein kinases in the livers from mice with MASH-HCC. Figure S2. Analysis of hepatic EpCAM and GRP78 abundance in mice with MASH-HCC. Figure S3. Analysis of hepatic glycogen levels in mice with MASH-HCC. Figure S4. Flow cytometric assessment of hepatic immune composition in mice with MASH-HCC. Figure S5. Analysis of hepatic p62 abundance in mice with MASH-HCC. Figure S6. Analysis of relative LC3-II to LC3-I ratio in the livers from mice with MASH-HCC. Figure S7. Comparison of whole-cell proteomic profiles between MST3 knockout and wild-type Huh7 cells. Figure S8. Analysis of relative phospho-STAT3 to STAT3 ratio in the livers from mice with MASH-HCC. [file 12916_2026_4812_MOESM3_ESM.pdf]

Supplementary Figure S1

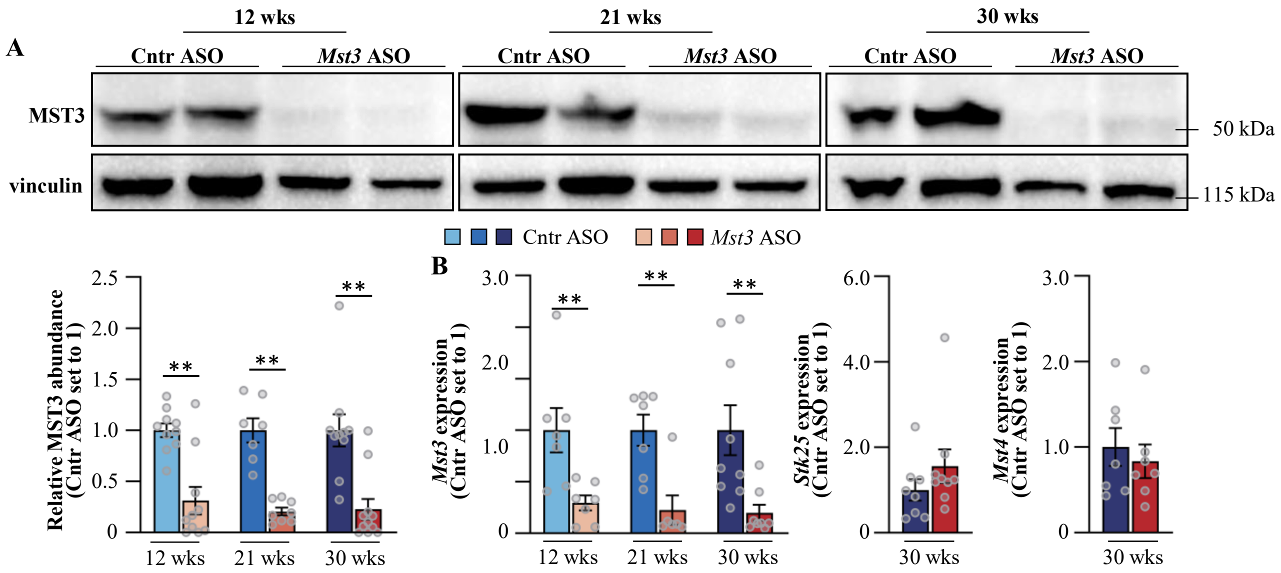

Supplementary Figure S1. Expression of MST3 and related protein kinases in the livers from mice with MASH-HCC. (*A*) Western blot analysis of MST3 in liver lysates. Representative Western blots are shown with vinculin used as a loading control. Quantification by densitometry is shown below. (*B*) Relative hepatic mRNA expression of *Mst3*, *Stk25*, and *Mst4* determined by RT-qPCR. Data are mean ± SEM from 6 to 11 mice per group. Cntr, control; wks, weeks. ***p* < 0.01.

Supplementary Figure S2

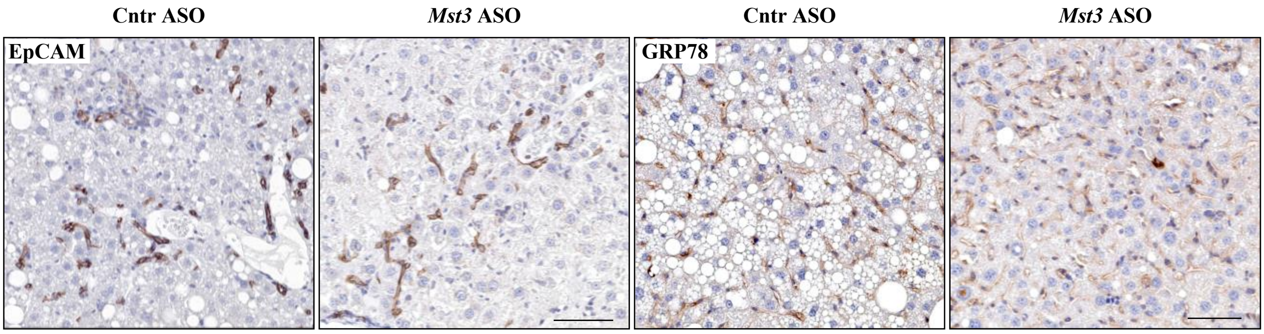

Supplementary Figure S2. Analysis of hepatic EpCAM and GRP78 abundance in mice with MASH-HCC. Liver samples were collected from mice treated with *Mst3* ASO or control ASO for 30 weeks. Representative images of liver sections processed for immunohistochemistry with anti-EpCAM (brown) or anti-GRP78 (brown) antibodies, counterstaining with hematoxylin. Scale bar, 100 µm. Cntr, control.

Supplementary Figure S3

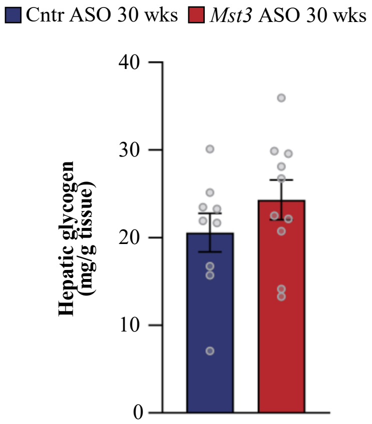

Supplementary Figure S3. Analysis of hepatic glycogen levels in mice with MASH-HCC. Liver samples were collected from mice treated with *Mst3* ASO or control ASO for 30 weeks. Data are mean ± SEM from 9 to 10 mice per group. Cntr, control; wks, weeks.

Supplementary Figure S4

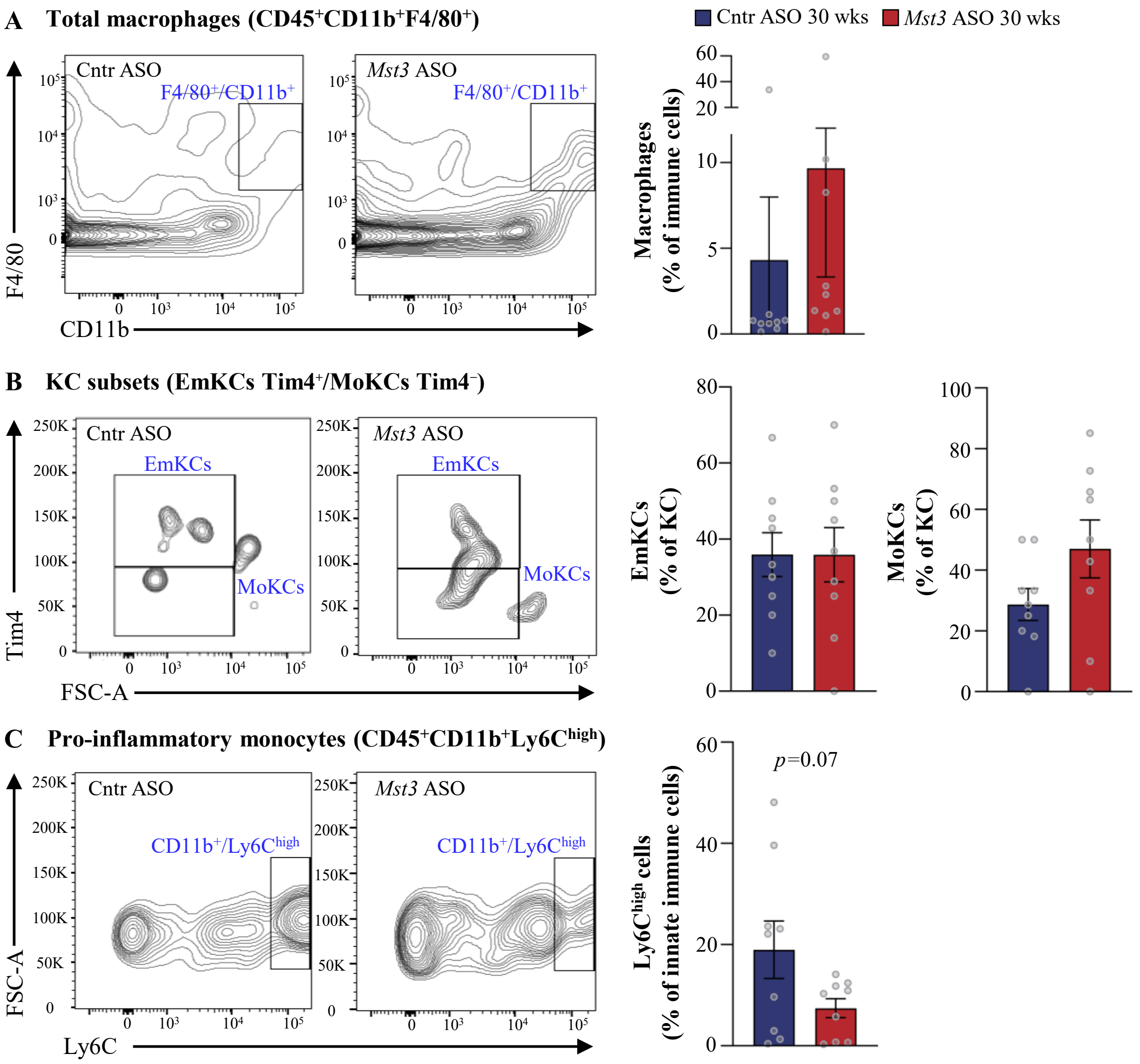

Supplementary Figure S4. Flow cytometric assessment of hepatic immune composition in mice with MASH-HCC. Liver samples were collected from mice treated with *Mst3* ASO or control ASO for 30 weeks. Liver immune cells were gated as singlets, and $CD45^+$ leukocytes, from which total macrophages, Kuppfer cell subsets, and pro-inflammatory monocytes were identified. (*A-C*) Representative flow cytometry plots and quantification of total macrophages ($CD45^+CD11b^+F4/80^+$) (*A*); Kuppfer cell subsets within the $Clec4F^+$ population including embryonically derived Kupffer cells ($Tim4^+$) and monocyte-derived Kupffer cells ($Tim4^-$) (*B*); and pro-inflammatory monocytes ($CD45^+CD11b^+Ly6C^{high}$) (*C*). Black boxes indicate the gates used for sorting and quantification. Data are mean ± SEM from 8 to 9 mice per group. Cntr, control; KC, Kupffer cells; EmKCs, embryonically derived Kupffer cells; MoKCs, monocyte-derived Kupffer cells, wks, weeks.

Supplementary Figure S5

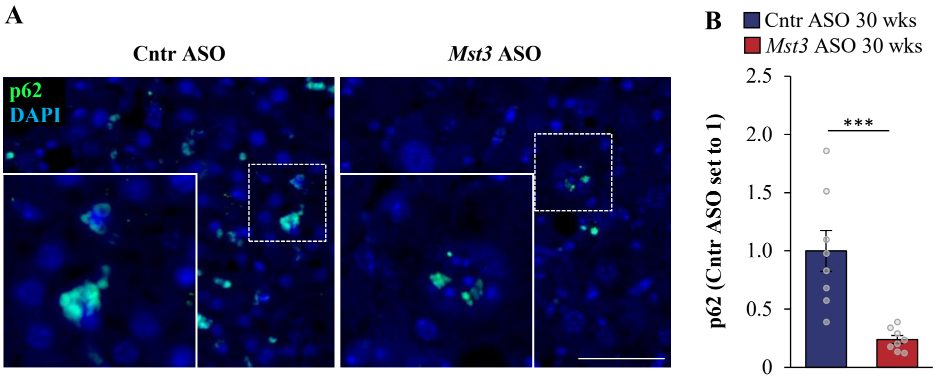

Supplementary Figure S5. Analysis of hepatic p62 abundance in mice with MASH-HCC. Liver samples were collected from mice treated with *Mst3* ASO or control ASO for 30 weeks. (*A*) Representative images of liver sections processed for immunofluorescence with anti-p62 (green) antibody; nuclei stained with DAPI (blue). Scale bar, 100 µm. (*B*) Quantification of fluorescence-positive area. Data are mean ± SEM from 8 mice per group. Cntr, control; wks, weeks. ***$p < 0.001$.

Supplementary Figure S6

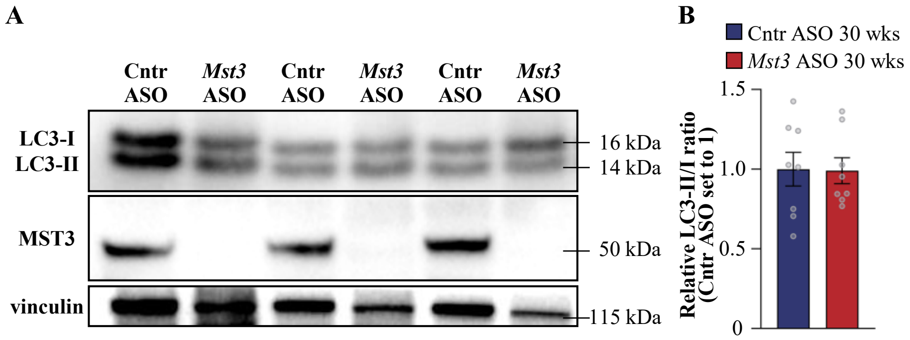

Supplementary Figure S6. Analysis of relative LC3-II to LC3-I ratio in mice with MASH-HCC. Liver samples were collected from mice treated with *Mst3* ASO or control ASO for 30 weeks. (*A*) Western blot analysis of LC3 and MST3 in liver lysates. Representative Western blots are shown with vinculin used as a loading control. (*B*) Quantification of LC3-II to LC3-I ratio by densitometry. Data are mean ± SEM from 8 mice per group. Cntr, control; wks, weeks.

Supplementary Figure S7

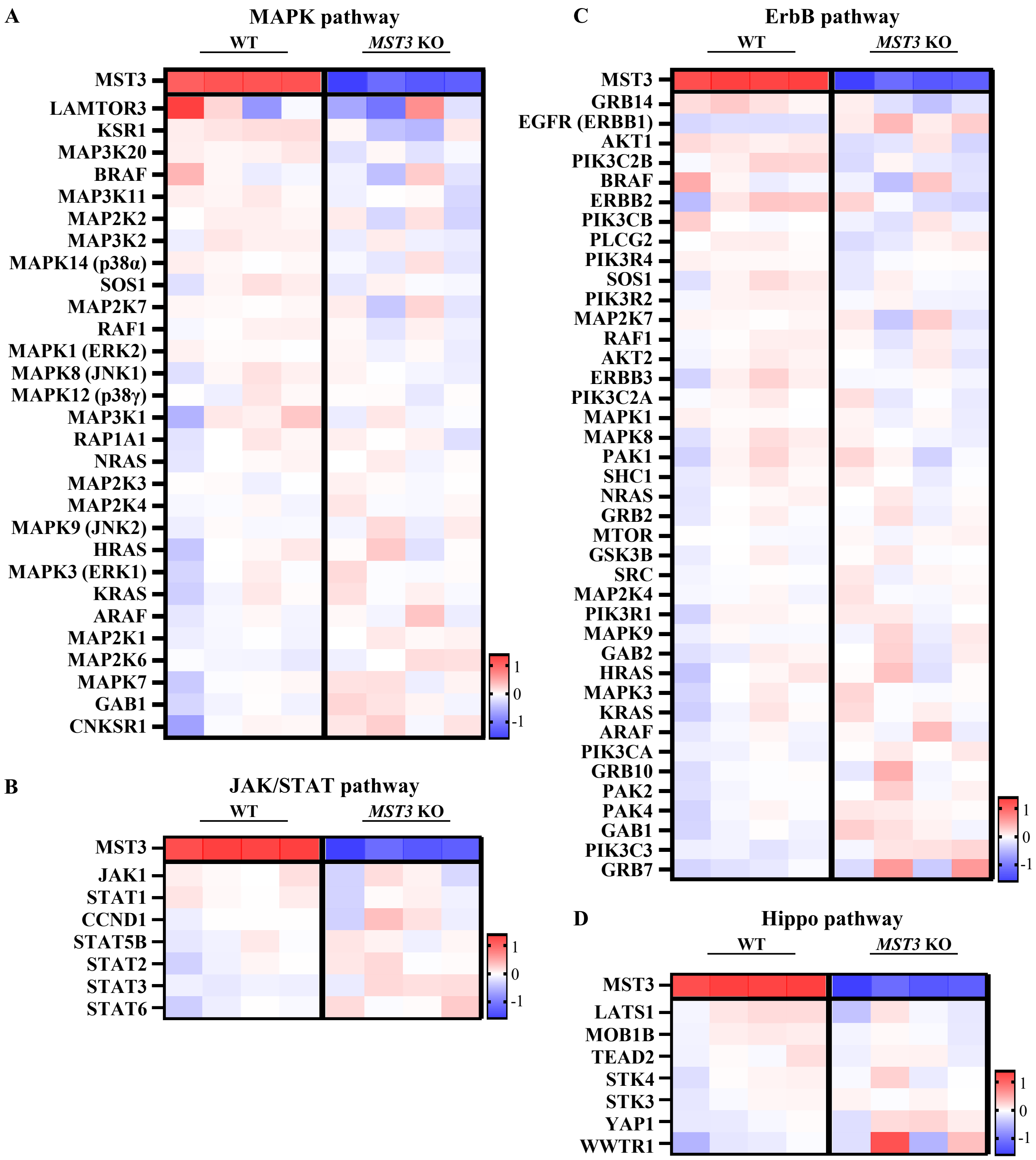

Supplementary Figure S7. Comparison of whole-cell proteomic profiles between *MST3* knockout and wild-type Huh7 cells. *(A-D)* Heat maps of a targeted, literature-guided evaluation of proteins with established roles in human HCC, including MAPK (*A*), JAK/STAT (*B*), ErbB (*C*), and Hippo (*D*) pathways. Applying this targeted approach, we found that only two proteins within the ErbB network were differentially expressed – EGFR was upregulated, while GRB14 was downregulated in MST3-deficient cells – and no coordinated alterations were detected across the examined pathways. The scaled abundance of four different clones per genotype is shown. The protein names are denoted on the left. KO, knockout; WT, wild-type.

Supplementary Figure S8

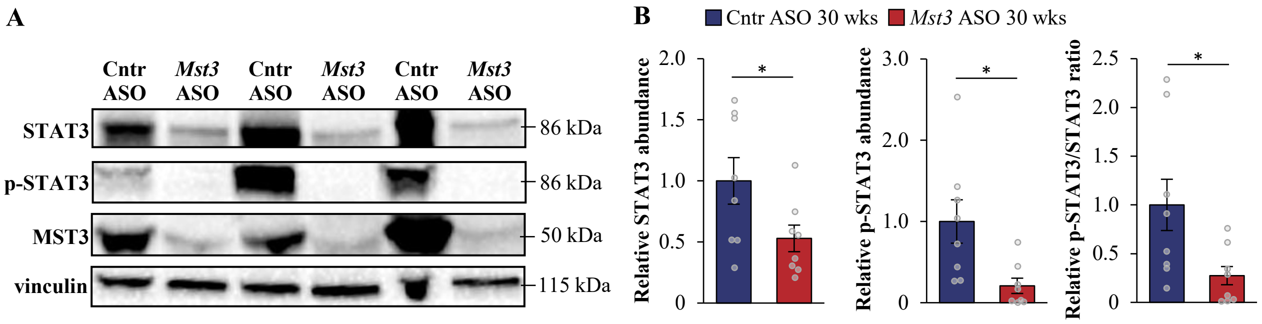

Supplementary Figure S8. Analysis of relative phospho-STAT3 to STAT3 ratio in mice with MASH-HCC. Liver samples were collected from mice treated with *Mst3* ASO or control ASO for 30 weeks. (*A*) Western blot analysis of STAT3, phospho-STAT3 (Tyr^{705}), and MST3 in whole liver lysates. Representative Western blots are shown with vinculin used as a loading control. (*B*) Protein levels quantified by densitometry. Data are mean ± SEM from 8 mice per group. Cntr, control; wks, weeks. *$p < 0.05$.
