# Supplementary material for: Targeting STE20-type kinase MST3 improves metabolic dysfunction-associated steatohepatitis without affecting hepatocellular carcinoma development in mice
Source: BMC Med. 2026 Mar 24;24:214. doi: 10.1186/s12916-026-04812-0 (PMC13063862; doi:10.1186/s12916-026-04812-0)

Additional File 4. Images of the original, uncropped Western blots

Targeting STE20-Type Kinase MST3 Improves Metabolic Dysfunction-Associated Steatohepatitis Without Affecting Hepatocellular Carcinoma Development in Mice

Jingjing Zhang, Xiangdong Gongye, Lohitesh Kovooru, Emma Andersson, Bernice Asiedu, Manoj Amrutkar, Nadia Gul, Caitlyn Myers, Sheri Booten, Dan Emil Lind, Ying Xia, Antonio Molinaro, Anetta Härtlova, Per Lindahl, Sue Murray, and Margit Mahlapuu

This Additional File includes images of the original Western blots corresponding to Supplementary Figures S1, S6, and S8. Full, uncropped membranes are provided. To facilitate clear viewing and correspondence with the representative immunoblots, all original Western blot bands presented in the supplementary figures have been marked with green boxes. Red crosses on page 2 indicate mice that were excluded from all downstream analyses due to insufficient ASO knockdown efficiency. Black vertical lines denote wells in which no protein was loaded.

Images of original Western blots represented in Supplementary Figure S1

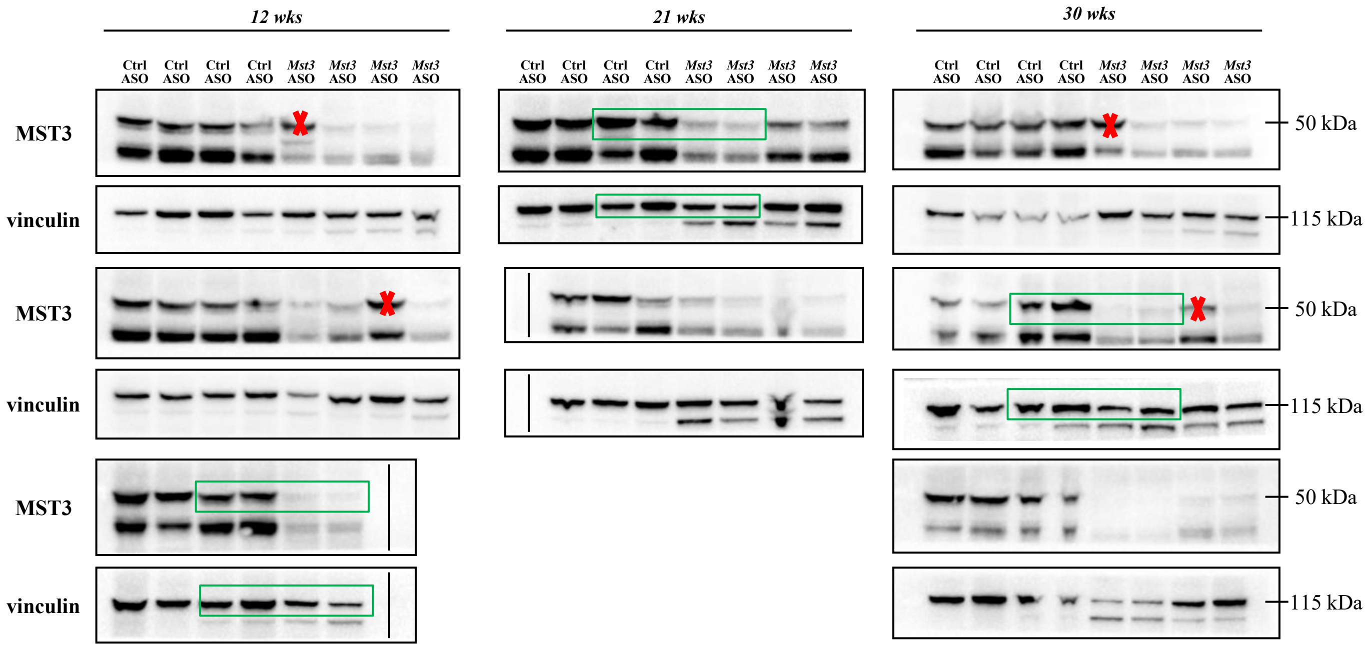

Images of original Western blots represented in Supplementary Figure S6

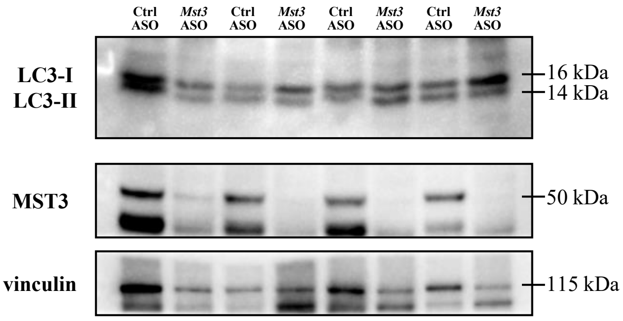

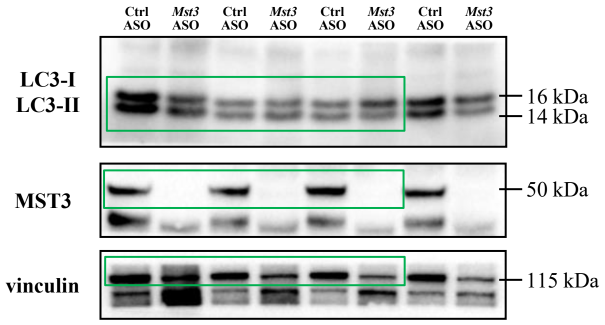

Images of original Western blots represented in Supplementary Figure S8

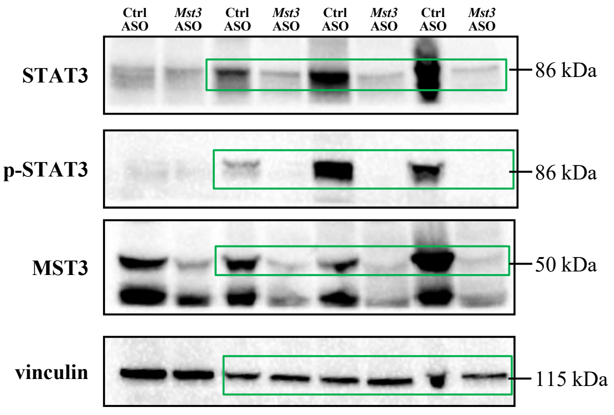

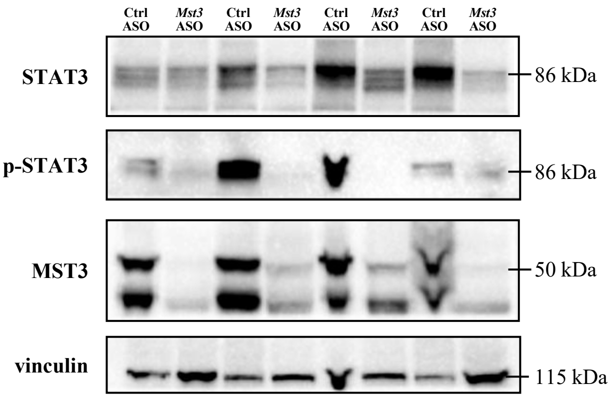

Supplement: Supplementary file 4 — Additional file 4: Images of the original, uncropped Western blots. [file 12916_2026_4812_MOESM4_ESM.pdf]
